# Anti-vascular agent Combretastatin A-4-P modulates Hypoxia Inducible Factor-1 and gene expression

**DOI:** 10.1186/1471-2407-6-280

**Published:** 2006-12-07

**Authors:** Gabi U Dachs, Andrew J Steele, Claudia Coralli, Chryso Kanthou, Andrew C Brooks, Sarah P Gunningham, Margaret J Currie, Ally I Watson, Bridget A Robinson, Gillian M Tozer

**Affiliations:** 1 Angiogenesis Research Group, Department of Pathology, Christchurch School of Medicine and Health Sciences, University of Otago, Christchurch, New Zealand; 2 Tumour Microcirculation Group, Gray Cancer Institute, Mount Vernon Hospital, Northwood, HA6 2JR, UK; 3 Academic Unit of Surgical Oncology, Division of Clinical Sciences, University of Sheffield, Sheffield, S10 2JF, UK

## Abstract

**Background:**

A functional vascular network is essential for the survival, growth and spread of solid tumours, making blood vessels a key target for therapeutic strategies. Combretastatin A-4 phosphate (CA-4-P) is a tubulin-depolymerising agent in Phase II clinical trials as a vascular disrupting agent. Not much is known of the molecular effect of CA-4-P under tumour conditions. The tumour microenvironment differs markedly from that in normal tissue, specifically with respect to oxygenation (hypoxia). Gene regulation under tumour conditions is governed by hypoxia inducible factor 1 (HIF-1), controlling angiogenic and metastatic pathways.

**Methods:**

We investigated the effect of CA-4-P on factors of the upstream and downstream signalling pathway of HIF-1 *in vitro*.

**Results:**

CA-4-P treatment under hypoxia tended to reduce HIF-1 accumulation in a concentration-dependent manner, an effect which was more prominent in endothelial cells than in cancer cell lines. Conversely, CA-4-P increased HIF-1 accumulation under aerobic conditions *in vitro*. At these concentrations of CA-4-P under aerobic conditions, nuclear factor κB was activated via the small GTPase RhoA, and expression of the HIF-1 downstream angiogenic effector gene, vascular endothelial growth factor (VEGF-A), was increased.

**Conclusion:**

Our findings advance the understanding of signal transduction pathways involved in the actions of the anti-vascular agent CA-4-P.

## Background

All solid tumours depend on a functional vascular supply for their growth, survival and metastatic spread [1]. Two new classes of anti-cancer agents specifically target the tumour blood vessels, namely anti-angiogenic agents, which interfere with the formation of new blood vessel, and anti-vascular agents, which target the existing tumour blood vessels (for a recent review see [2]). Combretastatin A-4 phosphate (CA-4-P), a tubulin-depolymerising agent structurally related to colchicines, is in clinical trials as a vascular disrupting agent. Phase I trials, started in 1998, have established a maximum tolerated dose (MTD) in the range of 52–68 mg/m^2^, and showed significant changes in tumour perfusion in patients by positron emission tomography (PET) and contrast-enhanced proton magnetic resonance imaging (MRI) at these doses [3-5]. CA-4-P has recently entered Phase II trials in combination with conventional radiation and chemotherapy [6].

Combretastatin A-4 was originally isolated from the South African bush willow *Combretum caffrum*. The phosphate derivative, CA-4-P, was subsequently synthesised as the more soluble prodrug [7]. The exact mechanism of action of CA-4-P is not yet understood. *In vitro*, CA-4-P causes microtubule depolymerisation, which leads to endothelial cell shape changes [8]. Other, more rapid effects include activation of the small GTPase Rho A and subsequent reorganization of the actin cytoskeleton, leading to an increase in endothelial monolayer permeability to macromolecules [9]. Rho A has been linked to the activation of transcription factors, including NF-κB [10].

Further work in endothelial cells demonstrated that CA-4-P damaged mitotic spindles, arrested cells at metaphase, and led to the death of mitotic cells with a characteristic G_2_/M DNA content, following prolonged drug exposures [11]. Mitotic arrest was associated with elevated levels of cyclin B1 protein and increased p34^cdc2 ^activity.

In animal models, CA-4-P caused a prolonged and extensive drop in blood flow in tumour vessels, with much less effect in normal tissues [12]. A rapid rise in tumour vascular permeability was observed leading to a catastrophic shutdown of the established tumour vasculature within minutes of drug exposure, causing necrosis and secondary tumour cell death [13].

CA-4-P treatment has been shown to increase tumour hypoxia (low oxygen) within one hour of drug treatment, as measured by Eppendorf oxygen electrode or with the use of the hypoxia marker pimonidazole [14,15]. Severe hypoxia is also known as an inherent characteristic of virtually all solid tumours, including experimental and clinical cancers, but not normal tissues. It has been correlated with poor prognosis, a more aggressive phenotype, increased metastases and resistance to therapy (see review by [16]). Early *in vitro *work had analysed CA-4-P cytotoxicity under 1% oxygen conditions (mild hypoxia), showing an increase in cell kill under hypoxia [17]. Beyond that, little is known of the effect of inherent tumour hypoxia on CA-4-P activity, or the effect of CA-4-P on gene expression.

Gene regulation under hypoxia is governed by the transcription factor, hypoxia inducible factor 1 (HIF-1) (for a review see [18]). HIF-1 is a heterodimer consisting of the subunits HIF-1α and HIF-1β/ARNT. Regulation involves ubiquitin-mediated, oxygen-dependent destruction of HIF-1α. The product of the von Hippel Lindau (vHL) tumour suppressor gene targets HIF-1α for oxygen-dependent proteolysis, acting as the substrate recognition component of an E3 ubiquitin ligase. In cancer, activity of the HIF system is up-regulated by microenvironmental hypoxia, growth factors and genetic changes, such as loss of vHL [19].

Since HIF-1 is the global hypoxic regulator of gene expression, and since CA-4-P both induces and acts under tumour hypoxia, we set out to determine the interactions of CA-4-P, hypoxia and HIF-1, as well as upstream and downstream factors of the HIF-1 signal transduction pathway.

## Methods

### Tissue culture

Human T24 bladder carcinoma and SW1222 colon carcinoma cells (American Type Culture Collection, Manassa, VA) were maintained in Dulbecco's modified Eagle's medium (DMEM, Life Technologies, Paisley, UK), 10% foetal calf serum (FCS) and glutamine (2 mM) in a humidified incubator, 37°C, in 5% CO_2_/air. Human umbilical vein endothelial cells from pooled donors (HUVECs, TCS CellWorks, Botolph Clayton, UK) were cultured on gelatine coated dishes in M199 media (Invitrogen, Paisley, UK) supplemented with 20% FCS, 4 mM L-glutamine, 20 μg/ml endothelial cell growth supplement (First Link, Birmingham, UK), and 80 μg/ml heparin. Only cells between the first and fourth passages were used for experiments. Human dermal microvascular endothelial cells (HDMECs, Clonetics, Cambrex, Wokingham, UK) were cultured in EBM-2 media (Clonetics) containing 5% FCS, hydrocortisone, hFGF-B, VEGF, IGF-1, ascorbic acid, hEGF and GA-1000 as supplied by the manufacturer (Clonetics).

Cells were treated and analysed when they either reached 40–50% confluence (sparse) or 80–90% confluence (dense). Sparse cells were actively dividing, whereas dense cells approached quiescence as determined by fluorescence activated cell sorting (FACS) analysis (results not shown). Densely growing endothelial cells were used to mimic quiescent, and possibly more mature vessels, whereas sparsely seeded cells were used as simple models to mimic the angiogenic phase of blood vessels. In a similar way, we studied sparsely and densely seeded tumour cell lines, as cell density has been implicated in gene expression studies in the past [20].

### HIF-1 induction

To achieve hypoxia and induce HIF-1, cells were plated in 6 cm oxygen impermeable Permanox dishes (Nalge, Nunc, Loughborough, UK) at 1–5 × 10^5^/dish (4–18 × 10^3^/cm^2^). Hypoxic conditions were achieved by placing the dishes in an anaerobic glove cabinet (in an atmosphere of 5% CO_2_, 5% H_2_, 90% N_2_, with palladium catalyst; Don Whitley Scientific LTD, Shipley, UK). The media pH was monitored and found to be the same in the aerobic incubator and the anaerobic glove cabinet. There was no significant difference is cell viability between aerobic and hypoxic cells over a 24 h period (results not shown).

Alternatively, the hypoxia-mimetic agent, CoCl_2 _(Sigma Aldrich, Gillingham, UK), was used at 100 μM concentration for varying periods of time 0–24 h [21]. Maximum HIF-1 accumulation was reached within 4 h of hypoxia or CoCl_2 _treatment, and stayed at that level for up to 24 h, and hence 4 h was used as the standard time for subsequent experiments.

### Drug treatment

CA-4-P was synthesised in house (Dr. M Naylor *et al*., Gray Cancer Institute; [7]) and used at 0.001, 0.01, 0.1, or 1 μM in complete medium, avoiding excessive exposure to light. The concentrations of CA-4-P used are clinically relevant (CA-4 peak plasma concentration at a dose of 68 mg/m^2 ^reached 2.26 μM; CA-4 concentration stayed above 0.01 μM in plasma for over 10 h in those patients [5]) and were shown to induce both morphological and functional changes in endothelial cells *in vitro *[9]. CA-4-P is the more soluble, phosphated, form of the active compound CA-4. CA-4-P is commonly used for *in vitro *and *in vivo *studies, where it is cleaved to the natural form by endogenous phosphatases and taken up into cells [22].

The Rho kinase inhibitors Y27632 (10 μM; Welfide Corporation (Osaka, Japan)) or HA1077 (10 μM; Calbiochem (Nottingham, United Kingdom)) were added to HUVECs for 1 hour prior to treatment with CA-4-P. HUVECs were incubated with the Rho A inhibitor C3 exoenzyme (10 μg/ml; Upstate (Dundee, Scotland)) for 12 hours before washing and treatment with CA-4-P. We have previously used these concentrations and exposure times, and shown them to be effective in preventing CA-4-P-induced cytoskeletal changes [9].

### Immunofluorescence

Cells were fixed and stained essentially as described previously [9]. Briefly, formalin-fixed and Triton-X-permeabalised cells were stained with primary antibody (Ab) (anti-HIF-1α at 1/250 (BD Biosciences, Cowley, UK), followed by secondary Ab (biotinylated anti-mouse, Vector Laboratories, Peterborough, UK) and FITC – Avidin (Vector laboratories) with DAPI (DNA stain, Vector Laboratories). Slides were visualised on a Nikon Eclipse TE200 Microscope.

### Western blotting

For HIF-1 detection, denatured total cell lysates were prepared as described previously [9]. Care was taken to include both adherent and floating cells, although few floating cells were observed. For NFκB studies, cellular fractions were isolated using a NE-PER kit (Perbio, Cramlington, UK) according to manufacturer's instructions. For Western blot analysis, equal amounts of protein (Pierce Micro BCA kit, Rockford, IL) were separated on NuPAGE 4–12% Bis-Tris Gels (Invitrogen) and transferred to nitrocellulose membranes. Anti-HIF-1α (primary Ab at 1/250, BD Biosciences) and secondary anti-mouse (HRP-labelled, Dako, Ely, UK) antibodies, or goat anti-human anti-NFκB (p65) antibody (Dako) and secondary anti-goat (HRP-labelled, Dako) were used. Immunoreactive bands were visualised by enhanced chemiluminescence (ECL, Amersham-Biosciences, Chalfont St Giles, UK). Equal protein loading was confirmed using EZBlue gel stain (Sigma). Normalisation using actin staining was not possible in this study, as CA-4-P acts directly on the actin cytoskeleton [9].

### Electrophoretic mobility shift assay (EMSA)

EMSAs were performed on nuclear fractions using an EMSA kit for NFκB (Panomics, Cambridge Biosciences, Cambridge, UK). Competition assays were performed in the presence of 100-fold excess of cold (unlabelled) NFκB probe. Supershift assays for p50 and p65 components were performed by incubation of samples with antibodies against p50 or p65 for 30 minutes prior to the addition of probe.

### Semi-quantitative real-time RT-PCR

RNA was extracted using Trizol (Invitrogen, Carlsbad, CA) following manufacturer's instructions. Total RNA (1 μg) was DNase-1 pre-treated (Invitrogen) immediately prior to reverse-transcription using a SuperScript™ First-Strand Synthesis System for RT-PCR (Invitrogen) according to manufacturer's instructions. Polymerase chain reaction (PCR) was carried out in a 25 μl reaction volume containing 1 μl of prepared cDNA, 10 μl RealMasterMix™ Probe (Eppendorf, Hamburg, Germany), 200 nM of each LUX FAM-labelled primer pair for the gene of interest (Invitrogen) and 100 nM of a commercial 18S LUX JOE-labelled primer (Invitrogen). Real-time RT-PCR was carried out in a Corbett Rotor-Gene 3000 thermal cycler (Mortlake, Australia). Cycling parameters consisted of an initial denaturation step at 95°C for 1 minute, followed by 45 cycles of denaturation at 95°C for 20 s, primer annealing at 63°C for 20 s with data acquisition, and extension at 68°C for 20 s, followed by melt curve analysis. Primer sequences for VEGF were as follows: Reverse primer (5'-3') CGA AGC CAT GAA CTT CAC CAC TT [FAM] G; forward primer (5'-3') GCT CTA CCT CCA CCA TGC CAA G.

### Statistical analysis

The mean levels were compared between groups using ANOVA. Where significant differences between groups were identified, these were further explored using Fisher's Least significant difference tests. A value of p < 0.05 was considered significant.

## Results

### Cellular localisation of HIF-1

**Figure 1:**
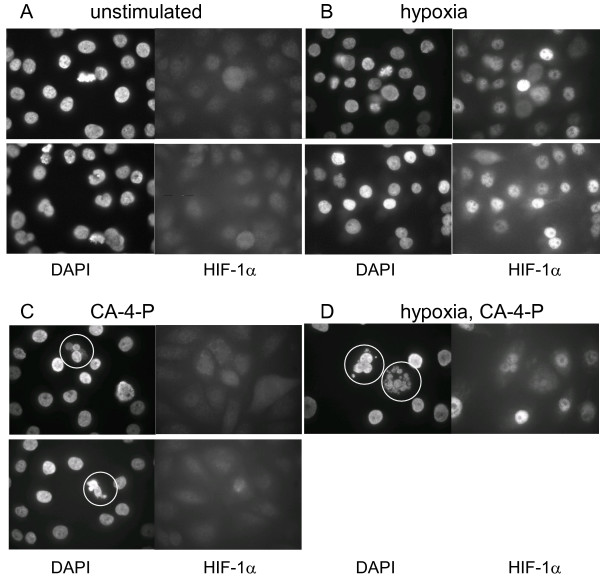
Effect of CA-4-P on cellular HIF-1 localisation and nuclear morphology. Immunofluorescent staining showed limited nuclear HIF-1 accumulation of unstimulated T24 cells (A) and CA-4-P-treated aerobic cells (C), with significant accumulation in hypoxic (B) and CA-4-P-treated hypoxic cells (D). CA-4-P treatment (1 μM) induced nuclear fragmentation (circled), but did not notably alter HIF-1 localisation. Identical microscopic fields of DAPI-stained nuclei (left) and HIF-1α stained cells (right) are shown.

Since translocation of the HIF-1 heterodimer to the nucleus is one of the steps characterising activation of HIF-1 [23], we analysed the cellular localisation of HIF-1α by immunofluorescence in T24 cancer cells (Figure 1). Immunofluorescence analysis demonstrated a variation in staining and nuclear localisation within the same cell population. Following stimulation by hypoxia or its mimetic agent, CoCl_2_, the tumour cells showed significant nuclear localisation of HIF-1α (Figure 1B). Treatment of hypoxic cells with CA-4-P (1 μM) did not appear to alter localisation of HIF-1 relative to hypoxia alone (Figure 1B,1D). Drug treated T24 cells showed signs of apoptosis (nuclear fragmentation) (Figure 1C,1D).

### HIF-1 accumulation following hypoxic CA-4-P treatment

**Figure 2:**
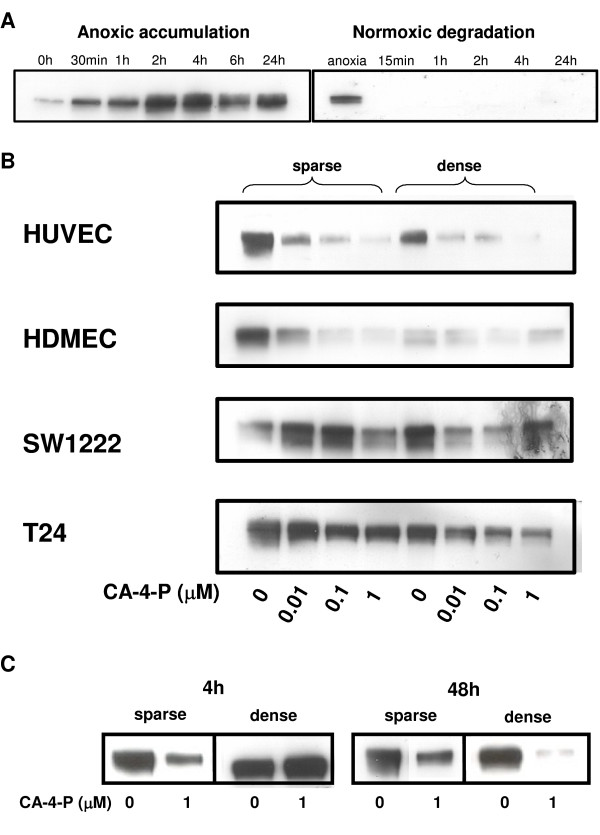
HIF-1 modulation by hypoxia and CA-4-P. **A**: Hypoxic accumulation and normoxic degradation of HIF-1.  Western blot analysis showed that T24 tumour cells exposed to increasing lengths of time under hypoxia accumulated HIF-1a, and HIF-1a degraded rapidly under aerobic conditions. **B**: Increasing concentrations of CA-4-P modulate the hypoxic accumulation of HIF-1. Sparse and dense endothelial and tumour cells were treated for 24h with increasing concentrations of CA-4-P (0 - 1mM), and subjected for the final 4h to hypoxia, prior to protein extraction and detection of HIF-1a by Western blot analysis. Similar results were obtained in >3 independent experiments; representative photographs are shown. **C**: Short pre-exposure to CA-4-P reduces hypoxic HIF-1 accumulation. T24 tumour cells exposed for 1h to CA-4-P showed a reduction in hypoxic HIF-1 accumulation 4h and 48h later, compared to untreated cells.

Using Western blot analysis, we confirmed that the HIF-1α subunit accumulated in T24 cells subjected to hypoxia or CoCl_2 _within 30 min and increased further up to a maximum at 4 h (remaining elevated up to 24 h), and degraded rapidly, within 15 min, following re-oxygenation (Figure 2A). Next, T24 and SW1222 cancer cells, as well as HUVEC and HDMEC endothelial cells *in vitro *were exposed to CA-4-P under hypoxia, and the effect on HIF-1 protein accumulation was analysed (Figure 2B). Western blot analysis of treated cell extracts indicated a trend towards a CA-4-P concentration- dependent reduction in HIF-1 accumulation under hypoxia. This effect was more prominent in the endothelial cells than the cancer cell lines. Also, the sparsely plated SW1222 colon cell line displayed some ambiguity in HIF-1 staining. Overall, the reduction in HIF-1 accumulation was seen whether cells were treated under hypoxia (Figure 2B) or with CoCl_2 _(results not shown), independent of cell density. This effect was evident whether cells were hypoxia-stimulated for 4 h at the start (results not shown) or end (Figure 2B) of the 24 h CA-4-P treatment period. A period of hypoxia (up to 24 h) did not change the number of viable or necrotic cells (trypan blue exclusion assays, results not shown).

To more closely mimic the clinical situation, we also analysed the effect of a short exposure to CA-4-P. CA-4-P in patients is rapidly converted to the active compound CA-4, which has a terminal plasma half-life of 3.3 h [24]. The Western blot data showed that even short (1 h) pre-exposure to CA-4-P under aerobic conditions reduced subsequent hypoxia-induced HIF-1 accumulation in T24 cells 48 h later (Figure 2C). A similar result was obtained at 4 h in sparsely but not densely plated cells.

### HIF-1 accumulation following normoxic CA-4-P treatment

**Figure 3:**
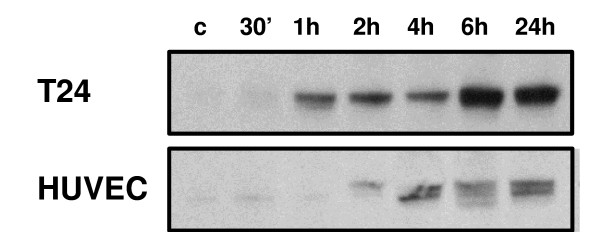
CA-4-P induces the accumulation of HIF-1 under aerobic conditions. Tumour and endothelial cells showed a time-dependent increase in HIF-1 accumulation under aerobic conditions during treatment with 1 μM CA-4-P. Similar results were obtained from 3 independent experiments; representative photographs are shown.

We exposed cells to 1 μM CA-4-P for varying periods from 30 min to 24 h, and observed an increase in HIF-1 accumulation under aerobic conditions (Figure 3). HIF-1 accumulation increased over time in both T24 tumour cells and HUVECs. Control cultures (without CA-4-P) showed no significant increase in HIF-1 accumulation over this time period (results not shown).

### NFκB activation via Rho A following CA-4-P treatment

**Figure 4:**
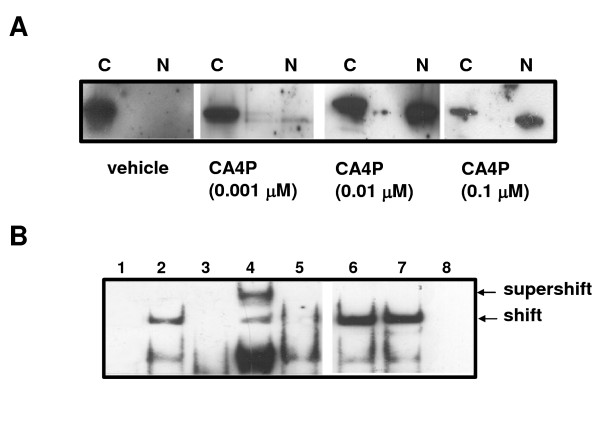
CA-4-P induces NFkB nuclear translocation and activation. **A**: CA-4-P induces NFkB translocation from the cytoplasm to the nucleus. Western blot analysis of CA-4-P treated aerobic HUVEC demonstrating   translocation of NFkB (p65) protein from the cytoplasmic to the nuclear   fraction within 1 h in a dose dependent manner. C: cytoplasmic fraction, N:   nuclear fraction. **B**: CA-4-P induces NFkB activation via Rho A, but not Rho kinase. EMSA showed that CA-4-P induced NFkB-DNA complex formation in aerobic   HUVEC. Supershift analysis using antibodies against NFkB components resulted in a further retardation of the p50 band and loss of the p65 band. Treatment with Rho kinase inhibitors (Y27632 and HA1077) demonstrated no effect on NFkB, whereas treatment with the Rho A inhibitor C3 prevented  NFkB activation by CA-4-P. (1) vehicle control, (2) 0.1mM CA-4-P, (3) +   cold probe, (4) + p50 Ab, (5) + p65 Ab, (6) + Y27632, (7) + HA1077, (8) + C3.

In order to determine the upstream signalling pathway involved in the aerobic stabilisation of HIF-1, we analysed NFκB activation by CA-4-P. Western blot analysis demonstrated that incubation of HUVEC with CA-4-P caused a dose dependent transfer of the p65 component of NFκB from the cytoplasmic fraction to nuclear fraction within 1 h (Figure 4A). Further analysis, using electrophoretic mobility shift assays (EMSA), demonstrated little constitutive binding of control nuclear extracts to an oligonucleotide containing the NFκB consensus sequence (Figure 4B). Following treatment with CA-4-P, two densely labelled complexes were observed (Figure 4B). These bands could be competed out by an excess of unlabeled (cold) NFκB probe. NFκB-DNA complex formation in response to CA-4-P appeared to peak at one hour, and was reduced to near control levels four hours post stimulation (results not shown). We performed Supershift assays using specific antibodies to p65 and p50 to reveal the identities of the protein in the CA-4-P-induced NFκB binding complex. Incubation with antibody to p50 or p65 resulted in a further shift of the p50-DNA complex, and the disappearance of the p65-DNA complex (Figure 4B), indicating that CA-4-P activates NFκB in the form of a p50·p65 heterodimer in HUVEC.

Incubation of HUVEC with the Rho A inhibitor C3 exoenzyme prevented the formation of CA-4-P-induced complexes. However, incubation of HUVEC with the Rho kinase inhibitors Y27632 and HA1077, prior to treatment with CA-4-P, had no effect on the formation of the NFκB complex (Figure 4B).

### VEGF induction following normoxic CA-4-P treatment

**Figure 5:**
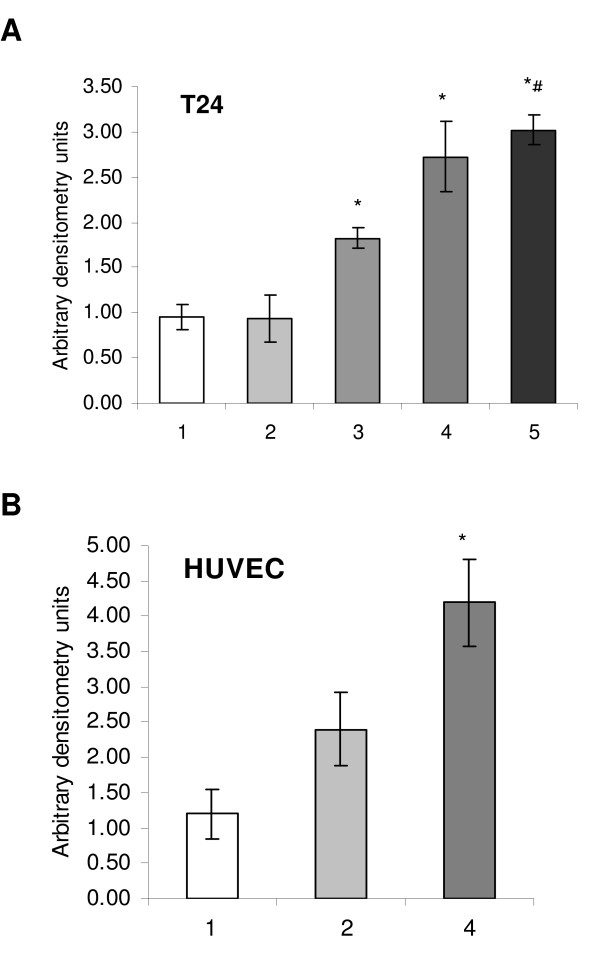
CA-4-P-modulation of the angiogenic growth factor VEGF. Expression of VEGF was detected by real time RT-PCR in T24 bladder carcinoma (A) and HUVEC (B) following aerobic treatment: (1) control, (2) 2 h CA-4-P (1 μM), (3) 24 h CA-4-P (1 μM), (4) 4 h CoCl_2 _(100 μM), (5) 24 h CA-4-P and 4 h CoCl_2_. RNA was extracted from two flasks each, and > 3 independent real time PCR reactions were performed on each; average and SEM are shown; * p < 0.05 compared to control; # p < 0.05 compared to (3).

In order to determine whether the observed CA-4-P-induced modulation of HIF-1 accumulation had an effect on down-stream HIF-1-regulated genes, we analysed the expression of vascular endothelial growth factor (VEGF-A) by real time RT-PCR. As expected, T24 cells and HUVECs treated with 100 μM CoCl_2 _showed a significant increase in VEGF expression of 2.9 and 3.5-fold, respectively, compared to aerobic controls (Figure 5). Cells exposed to CA-4-P under aerobic conditions for 24 h (T24, p < 0.05) or 2 h (HUVEC, p < 0.05) showed an increase in VEGF expression of 1.9 and 2.0-fold, respectively, reflecting HIF-1 accumulation under aerobic conditions. T24 cells exposed to CA-4-P for only 2 h, however, showed no modulation of VEGF expression (Figure 5A). The combination of CA-4-P for 24 h and CoCl_2 _for 4 h in T24 cells did not increase VEGF above CoCl_2_-treatment alone (Figure 5A)

## Discussion

We analysed the effect of CA-4-P on the transcription factor HIF-1, as well as members of its upstream and downstream signal transduction pathway *in vitro*. Our results have demonstrated that CA-4-P could differentially modulate members of the HIF-1 signal transduction pathway depending on the cell's oxygenation status. Although the findings require further systematic follow-up, they are valuable for generating new hypotheses.

A trend towards a CA-4-P-concentration dependent reduction in hypoxic HIF-1 accumulation was observed. The significance of these *in vitro *findings is not yet clear. However, these results have some anecdotal support from a preliminary *in vivo *study indicating that therapeutic CA-4-P doses reduced HIF-1 accumulation in SW1222 xenografted tumours at 24 h but increased HIF-1 at 4 h after treatment (results not shown).

The preliminary *in vivo *data can be explained by a simple model, whereby acute hypoxia is caused by CA-4-P-induced-vascular disruption [14,15], resulting in the early accumulation of HIF-1, followed at later times by the loss of hypoxic cells by necrosis, with their accumulated HIF-1, resulting in an apparent reduction in HIF-1 protein. Further *in vivo *work is clearly required.

There also appeared to be a more prominent effect in endothelial cells compared to tumour cells. Endothelial PER-ARNT-SIM domain protein (EPAS), also known as HIF-2α, has 48% sequence identity to HIF-1α, and was initially shown to be limited to the endothelium of mouse embryos [25]. Since then, both HIF-1α and HIF-2α were detected in most human cells, and both activate a range of hypoxia response elements with similar efficacy. Importantly for our research, a study using short interfering RNAs against the two HIFs established that in endothelial cells, hypoxia-inducible gene expression of a panel of genes was critically dependent only on HIF-1, and not HIF-2 [26]. Hence, our result demonstrating CA-4-P modulation of HIF-1α in endothelial cells is relevant. In addition, we have found no cell type or condition-specific accumulation of HIF-2α across our panel of cells and cell lines (results not shown).

Our findings, of an increase in HIF-1 in response to normoxic CA-4-P treatment, agree with data from Jung *et al *[27] who used the microtubule disrupting agents colchicine (1 μM), vinblastine (0.1 μM) and nocodazole (10 μM). That group showed that HIF-1 induction was dependent on transcription and on activation of NFκB. Our work supports their data by demonstrating translocation and subsequent binding of NFκB to specific DNA sequences following CA-4-P treatment. Hence it is likely that CA-4-P induction of HIF-1 also acts through NFκB, similar to other tubulin disrupting agents. Our data further show that NFκB activation by CA-4-P was dependent on Rho A. This link is supported by previous work using thrombin showing a direct signal transduction via Rho A to NFκB [28]. However, in contrast to our previous findings for other signalling pathways, which showed a role for Rho kinase in CA-4-P induced effects [9], CA-4-P-induced NFκB activation was shown to be independent of Rho kinase. This suggests that other downstream effectors of Rho A may be involved in NFκB activation.

Our results demonstrated that VEGF expression mirrored CA-4-P-induced HIF-1 accumulation under aerobic conditions, indicating that the accumulated HIF-1 is active and able to regulate transcription of downstream genes. VEGF is the most potent angiogenic growth factor in solid tumours. It also has a range of other functions, including induction of vascular permeability and supporting survival of endothelial cells. Although the vasculature of solid tumours is known to be leaky, a further increase in vascular permeability due to CA-4-P treatment has been demonstrated *in vivo *[13]. Our findings in this paper are consistent with published data on another combretastatin family member, OXI4503 [29], showing that elevated tumour vessel permeability was accompanied by an increase in VEGF from 1 hr post drug treatment *in vivo*. Hence, our observed increase in VEGF expression and HIF-1 accumulation under aerobic conditions with clinically relevant concentrations of CA-4-P may in part explain previous *in vivo *findings. It is interesting to note that in our work on VEGF expression, endothelial cells seemed to respond earlier than cancer cells, and this finding requires further investigation.

## Conclusion

HIF-1 protein has been reported in all human tissue and organs assayed, and shown to be over-expressed in many human tumours, including breast, bladder and colon carcinomas [19,30]. Our data has shown that CA-4-P affects members of the HIF-1 signal transduction pathway. There appears to be support for both increases and decreases in HIF-1 accumulation *in vitro*, and both scenarios have possible implications for tumour biology, as HIF-1 is the main regulator of a range of angiogenic, vasoactive and metastatic factors [16]. The impact of these findings on treatment response, if any, remains to be determined.

## Competing interests

The author(s) declare that they have no competing interests.

## Authors' contributions

GUD conceived and designed the study, and drafted the manuscript. GUD, CC, AJS and AIW carried out the cell culture and Western blotting studies. ACB did the gel shift experiments, and SPG carried out the RT-PCR studies. MJC, BAR and GMT critically revised the manuscript, and GUD gave approval of the final version. All authors read and approved the final manuscript.

## Pre-publication history

The pre-publication history for this paper can be accessed here:
